# Chitosan Contribution to Therapeutic and Vaccinal Approaches for the Control of Leishmaniasis

**DOI:** 10.3390/molecules25184123

**Published:** 2020-09-09

**Authors:** Philippe M. Loiseau, Sébastien Pomel, Simon L. Croft

**Affiliations:** 1 Antiparasite Chemotherapy, CNRS, BioCIS, Université Paris-Saclay, 92290 Châtenay-Malabry, France; sebastien.pomel@u-psud.fr; 2 Faculty of Infectious and Tropical Diseases, London School of Hygiene & Tropical Medicine, London WC1E 7HT, UK; simon.croft@lshtm.ac.uk

**Keywords:** chitosan, drug carriers, leishmaniasis, chemotherapy, vaccine

## Abstract

The control of leishmaniases, a complex parasitic disease caused by the protozoan parasite *Leishmania*, requires continuous innovation at the therapeutic and vaccination levels. Chitosan is a biocompatible polymer administrable via different routes and possessing numerous qualities to be used in the antileishmanial strategies. This review presents recent progress in chitosan research for antileishmanial applications. First data on the mechanism of action of chitosan revealed an optimal in vitro intrinsic activity at acidic pH, high-molecular-weight chitosan being the most efficient form, with an uptake by pinocytosis and an accumulation in the parasitophorous vacuole of *Leishmania*-infected macrophages. In addition, the immunomodulatory effect of chitosan is an added value both for the treatment of leishmaniasis and the development of innovative vaccines. The advances in chitosan chemistry allows pharmacomodulation on amine groups opening various opportunities for new polymers of different size, and physico-chemical properties adapted to the chosen routes of administration. Different formulations have been studied in experimental leishmaniasis models to cure visceral and cutaneous leishmaniasis, and chitosan can act as a booster through drug combinations with classical drugs, such as amphotericin B. The various architectural possibilities given by chitosan chemistry and pharmaceutical technology pave the way for promising further developments.

## 1. Introduction

Leishmaniases are neglected tropical and sub-tropical diseases with an estimated 0.7 to 1 million new cases per year in nearly 100 endemic countries, caused by *Leishmania* spp., a protozoan parasite transmitted by the female Phlebotomine sandfly [1,2]. About 20 *Leishmania* species are able to infect humans and two main clinical manifestations are usually described: visceral leishmaniasis (VL), which is fatal in the absence of treatment and also affects dogs, and cutaneous leishmaniasis (CL), which is self-curing but leads to disfigurement and stigmatisation. There are other clinical manifestations of CL including mucocutanous and diffuse forms. Whereas some vaccines exist for dogs—with uncomplete efficacy—none are marketed for human use. Chemotherapy is presently the single approach to manage these diseases, in combination with a more intense vector control [1]. The current treatment recommended for leishmaniases include the pentavalent antimonials (Glucantime^®^ and Pentostam^®^), liposomal amphotericin B (AmBisome^®^; LampB), paromomycin used by parenteral route, and miltefosine (Impavido^®^), the latter remaining the single orally active drug [3]. However, most of these drugs have limitations of high cost, significant adverse effects, variable effectiveness and drug resistance (for the antimonials for VL in the Indian subcontinent [ISC]); only LampB currently donated via WHO for VL in a single course treatment in the ISC approaches an acceptable treatment. Therefore, there is a real need for safe, efficient, affordable, short course, oral, and field-adapted drugs for the treatment of leishmaniases.

However, the intracellular habitat of the amastigote stage of the *Leishmania* parasite is a challenge for the treatment of leishmaniases as the drug candidates have to accumulate in the phagolysosomal compartment of the host macrophages and selectively kill the parasite without cytotoxic effects. Different approaches have been developed to discover novel chemical entities active against the intracellular amastigote including: (i) systematic in vitro high-through-put screening of numerous chemical libraries, (ii) exploration of specific validated biochemical targets, (iii) immunomodulatory and other host-related strategies, (iv) drug combinations with drugs exhibiting different mechanisms of action, and (v) formulations to enhance the bioavailability of the classical drugs [4,5,6,7,8], including topical formulations for CL that might also reduce adverse effects [9]. Chitosan, a biodegradable cationic polysaccharide, which has direct anti-leishmanial activity, immunomodulatory activity and can be used as a drug delivery vehicle, has proved to be attractive subject for studies on leishmaniasis. 

**Figure 1:**
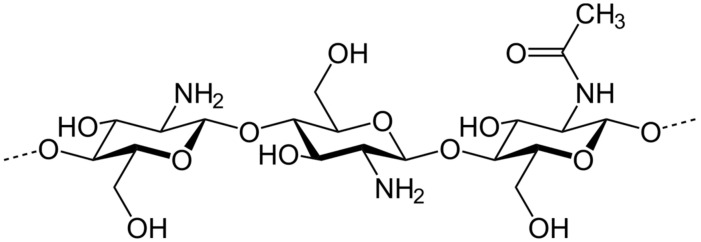
Chemical structure of chitosan polymer.

The chemical structure of chitosan is presented in Figure 1.

This review aims to present the most recent findings from the literature considering chitosan and its derivatives in different formulations for both therapeutic and vaccine purposes in the treatment and prevention of both cutaneous and visceral leishmaniasis.

Table S1 presents the main characteristics of the chitosan-based systems that have been developed these past years. Table S2 presents the antileishmanial and cytotoxic properties of the chitosan-based systems also developed these past years.

## 2. Chitosan: Nature and Different Uses

Chitosan is a linear polysaccharide composed of randomly distributed β-(1→4)-linked D-glucosamine and *N*-acetyl-d-glucosamine, as presented in Figure 1. It is obtained from the treatment of the chitin shells of shrimp and other crustaceans with an alkaline substance, such as sodium hydroxide leading to a deacetylation process. It can also be obtained from fungal source. The percentage of deacetylation can be determined by NMR spectroscopy, and it ranges from 60% to 100% in commercial chitosans. The molecular weight of commercially produced chitosan ranges from 3800 to 20,000 Daltons. Chitosan’s amino group has a pK_a_ value of ~6.5, leading to significant protonation at neutral pH, increasing with decreased pH and the percentage of deacetylation. This property makes chitosan both water-soluble and bioadhesive, easily binding to negatively charged surfaces such as mucosal membranes [10]. It is a biocompatible and biodegradable polymer that significantly enhances the transport of polar drugs across epithelial surfaces [11]. Chitosan has a large number of commercial and possible uses. In agriculture, chitosan can act as a seed treatment and biopesticide, helping plants to control fungal infections and it is also used in winemaking to prevent spoilage [12,13]. In the field of medicine, chitosan is used in bandages to reduce bleeding [14].

Chitosan is also proposed as a potential anti-infective agent. Thus, low molecular weight chitosan has been shown to exhibit antiviral, antibacterial, and antifungal activities [15,16,17]. Among medical applications, the search for anti-infective agents to treat diseases caused by drug resistant microorganisms has been gaining increased attention. The addition of hydrophobic chains onto the chitosan molecule by the reaction of N-acylation with maleic anhydride, making it amphiphilic, highly improved its antimicrobial activity by increasing electrostatic interaction with the cell walls of *Staphylococcus aureus* and *Escherichia coli* [18]. However, no activity of this modified polymer was detected against the protozoan *Leishmania amazonensis*. As no cytotoxicity was observed, this system appeared to be promising for further studies against bacteria [18]. In contrast, C-6 oxidized chitosan derivative obtained from chemical chitosan oxidation that had been degraded by enzymes systems such as endocellulase, hyaluronidase, hyaluronate lyase, chitinase, and other proteins, exhibited low antileishmanial activity against *Leishmania infantum* LIPA 137 with an IC_50_ value at 125 µg/mL, whereas the antibacterial activity was not significant at all [19]. These data highlight some clues about how chemical modifications of the chitosan skeleton enhance its antileishmanial activity. In addition, the immunostimulatory activity of chitosan with its capacity to induce Th1 cell responses could also be valorized in the field of antileishmanial chemotherapy [20]. According to the Food Drug Association (FDA), chitosan is a non-toxic, biodegradable, and biocompatible substance [21,22,23,24]. This enables chitosan also to be used to as drug delivery vehicle, either parenterally or integrated in drug formulations for topical use [25]. Within this latter context, the added value of chitosan would also be the acceleration of wound healing [26]. Such complementary and convergent properties could possibly help reduce the disfiguring leishmaniasis scars post-leishmaniasis. Topical therapy is an ideal treatment for simple CL because of the ease of administration and lower cost. Thus, chitosan or chitosan derivatives could be part of a suitable mono or bi/tri-therapy for localized cutaneous leishmaniasis or in combination with systemic therapies for more severe forms of the disease.

## 3. Activity of Chitosan and Its Derivatives on *Leishmania* Parasites

### 3.1. In Vitro Antileishmanial Activity of Chitosan and Its Derivatives

Several studies have described the in vitro activities of chitosan against *Leishmania* sp. Thus, low molecular weight chitosan, with 95% degree of deacetylation, was completely effective at concentrations of 100 μg/mL on promastigotes of *L. major* after 180 min of application [27]. In vitro activities of chitosan and some of its derivatives have also been studied in depth against *L. major* and *L. mexicana*, showing that the pH of the culture medium is critical for the activity against both promastigotes and intramacrophage amastigotes [28]. Thus, chitosan and its derivatives appeared approximately 7 to 20 times more efficient at pH 6.5 than at pH 7.5, and high-molecular-weight chitosan was the most active. This activity is positively correlated with the level of protonation of the polymer, allowing a better interaction with the negatively charged parasite membranes. Although the production of nitric oxide and reactive oxygen species was stimulated by high-molecular-weight chitosan in both uninfected and *Leishmania*-infected macrophages in a time- and dose-dependent manner at pH 6.5, the antileishmanial activity of chitosan was found not to be mediated by these metabolites. Analysis of the mechanism of action, based upon confocal imaging showed that rhodamine-labeled chitosan was taken up by pinocytosis and accumulated in the parasitophorous vacuole of *Leishmania*-infected macrophages [28]. This is the first demonstration of the mechanism of chitosan uptake by *Leishmania*-infected macrophages and of its location within the parasitophorous vacuole. Therefore, intrinsic antileishmanial activity of chitosan and its previously described immunodulatory effect are promising for further exploration of the capacity of this polymer to cure infected animals alone or in combination with antileishmanial drugs.

### 3.2. In Vivo Antileishmanial Activity of Chitosan on Cutaneous Leishmaniasis BALB/c Mice Model

Due to its biocompatibility, biodegradability, non-toxicity, and antimicrobial activity, chitosan is one of the most investigated biopolymers for wound healing [29,30,31]. A wide variety of biomedical applications have been reported for chitosan, especially for the treatment of cutaneous leishmaniasis. A study was performed in BALB/c mice to evaluate the efficacy of nanochitosan films in the treatment of cutaneous leishmaniasis caused by an Iranian strain of *Leishmania major* [32]. The application of nanochitosan film increased the reepithelialization rate, wound contraction rate, and the scar tissue formation, and in combination with Glucantime^®^ significantly reduced both lesion size and parasite load. Nanochitosan films, when administered alone or in combination with Glucantime^®^ by the intraperitoneal route, were shown to enhance glutathione peroxidase (GPX) activity and decrease lipid peroxidation and oxidative stress [32]. In other studies, low molecular weight chitosan, with 95% degree of deacetylation, significantly reduced the mean size of dermal lesions in BALB/c mice infected with an Iranian strain of *L. major* after a topical treatment at 200 and 400 µg/mL for 28 days compared to the control group [27].

### 3.3. Pilot Clinical Study of Chitosan Efficacy on Cutaneous Leishmaniasis Lesions in Patients

After confirming the effectiveness of nano-chitosan films in the treatment of cutaneous leishmaniasis in susceptible laboratory animals, a pilot clinical study was performed in 10 patients, in the absence of a control group [33]. The safety and efficacy of a chitosan-based biocompatible dressing was determined in patients affected by cutaneous leishmaniasis who were either unresponsive to or who presented medical contraindications to standard treatments. The film was maintained over the wound site for one week and was renewed every week until the healing was completed. All patients showed either significant (30%) or complete (70%) re-epithelialization of the skin lesion as well as microscopic negative results for amastigote forms of *Leishmania* after eight weeks of therapy. It is notable that all cases were completely cured at 16 weeks post treatment, in the absence of allergic reaction or infection. The fact that no failure was monitored in any patients after six months follow-up, makes this a promising approach to therapy [33]. However, further investigations need to be carried out by including a randomized double-blinded clinical trial with more patients.

## 4. Chitosan-Based Drug Loaded Formulations for the Chemotherapy of Cutaneous Leishmaniasis

### 4.1. Amphotericin B-Chitosan Nanoformulations

Amphotericin B (AmB) is an active agent against leishmaniasis, but its use is hampered by its high toxicity. Although amphotericin B-deoxycholate (AmB-DOC) showed its ability to partially cure the infection after intravenous administration, it can lead to dose-limiting side-effects mainly due to nephrotoxicity. The importance of AmB in the treatment of leishmaniasis, explains why AmB is the most studied drug to develop various strategies for bioavailability improvement.

A nano-sized chitosan AmB formulation was prepared by a phase separation method with a drug loading efficiency of 90% [34]. In vitro, a strong reduction of AmB cytotoxicity was observed with an improvement of activity. The slow drug release allowed a 98% cellular uptake of AmB. In vivo, a significant reduction of lesions along with complete wound healing was observed [34]. This formulation is worth further study. 

The evaluation of an AmB nanoparticles-delivery system, containing chitosan and chondroitin sulfate (NQC-AmpB) in a *Leishmania amazonensis* BALB/c mice model was combined with an in vivo biodistribution study showed that NQC-AmpB significantly reduced the lesion size and parasite burden with higher IFN-γ and IL-12 rates, and lower rates of IL-4 and IL-10, in comparison to the control groups. No significant toxicity was observed in the animals treated with NQC-AmpB at the doses used of 1 mg/kg of AmpB for 10 days [35]. These data should be confirmed by clinical trials, and the industrial scale-up with such a complex system should be compatible with limited cost of production.

Solid lipid nanoparticles (SLNs) have previously emerged as an interesting substitute to polymeric nanoparticles. SLNs loaded with AmB showed the advantage of combination between AmB chemotherapy with the immunomodulatory effect induced by chitosan [36]. Solvent emulsification and evaporation method were used to obtain uncoated and chitosan-coated AmB-loaded SLNs (AmB-SLNs). The internalization of chitosan-coated AmB-SLN by J774A.1 cells was more efficient than those with the uncoated formulation, without hemolytic effect. The in vitro antileishmanial activity of chitosan-coated AmB-SLN was higher than those of AmBisome^®^ and Fungizone^®^ [36]. TNF-α and IL-12 productions were stimulated by chitosan-coated AmB-SLNs. In addition, this formulation exhibited a better in vitro and in vivo safety profile than the commercial formulations. Therefore, AmB-SLNs have the required qualities to be evaluated in vivo on experimental leishmaniasis models.

A more complex system, consisting of self assembled sodium alginate cross-linked AmB loaded glycol chitosan stearate nanoparticles (AmB-SA-GCS-NP) was prepared using strong electrostatic interactions between oppositely charged polymer and copolymer using the ionotropic complexation method [37]. The nanocrystals obtained exhibited a size of 196 nm. Tagged FAmB-SA-GCS-NP had significantly higher (~1.7) uptake by J774A.1 cells in comparison to tagged FAmB. The in vitro antileishmanial activity, with IC_50_ values around 0.1 µg AmB/mL, and the in vivo reduction of parasite burden by 70% showed that AmB-SA-GCS-NP had significant improved activity over AmB alone. The decrease of in vitro and in vivo toxicities was ascribed to the monomeric form of AmB within SA-GCS-NP, compared to plain AmB [37]. AmB-SA-GCS-NP presents the advantage of a well-structured molecular organization leading to significant advantages in terms of activity and toxicity to be explored further. 

A mannose-anchored thiolated chitosan (MTC) amphotericin B nanocarrier was also developed and characterized to improve AmB biocompatibility and antileishmanial activities [38]. These nanoparticles were rod shaped with a size of ∼480 nm and elicited a 71-fold enhancement of drug uptake compared to native AmB in both uninfected and AmB sensitive *L. donovani*-infected macrophages. Morever, MTC nanocarriers retained and slowly released AmB over a 10-day period, whereas, with the native drug, no AmB was detected after two days of treatment. Macrophage survival was evaluated at 90% with MTC at the highest concentration of AmB tested (75 µg/mL) while it was only at 34.5% with the native drug under the same conditions. The MTC-AmB formulation exhibited a 10-fold increase of the in vitro antileishmanial activity compared to the native drug, with IC_50_ at 0.02 µg/mL and 0.26 µg/mL for MTC-AmB and AmB, respectively. Moreover, permeation of MTC-AmB across the Caco-2 cell monolayer was increased by 3-fold in comparison to pure AmB [39]. Pharmacokinetic studies have shown that MTC-AmB half-life and oral bioavailability were increased by 3.3- and 6.4-fold compared to pure AmB [39]. Moreover, oral administration of 50 mg/kg of MTC-AmB showed less hepato- and renotoxicity compared to AmB. Accordingly, in *L. donovani*-infected mice, a large reduction of parasite burden (89%) was observed after oral administration of 1 mg/kg MTC-AmB for seven days, in contrast to the native drug (17%). This MTC-AmB formulation is promising as an oral administration of AmB for leishmaniasis treatment. 

A chitosan-based formulation, using *N*-palmitoyl-*N*-methyl-*N*,*N*-dimethyl-*N*,*N*,*N*-trimethyl-6-*o*-glycol chitosan (GCPQ), was also used to encapsulate AmpB for oral treatments [40]. This chitosan derivative is a self assembling nanoparticle internalized by enterocytes enhancing the bioavailability of hydrophobic drugs [41,42]. The encapsulation of AmpB in GCPQ resulted in nanoparticles of a size of 216 and 35 nm, the bimodal size being due to an equilibrium established between drug filled particles and empty micelles. Besides an oral bioavailabity of AmB-GCPQ nanoparticle, evaluated at 24%, this oral formulation was shown to exhibit a comparable efficacy to parenteral AmBisome^®^ in the treatement of visceral leishmaniasis in vivo in *L. infantum* infected Balb/c mice, and also in murine models of candidiasis and aspergillosis. This formulation also paves the way for a new oral treatment of visceral leishmaniasis by using bioavailable AmB-encapsulated nanoparticles.

Chitosan nanoparticles modified with a ligand 4-sulfated N-acetyl galactosamine (4-SO4GalNAc), called SCNPs, were loaded with AmB producing an AmB-SCNPs formulation with a mean particle size of 333 nm, for an AmB improved delivery to infected macrophages [43]. In vitro studies using J774A.1 macrophages showed an enhanced uptake of AmB-SCNPs in comparison with AmB loaded unmodified chitosan NPs (AmB-CNPs). After intravenous administration to *L. donovani* infected hamsters at a dose equivalent of 1 mg/kg/day of AmB for two days, AmB-SCNPs allowed a higher AmB concentration within liver and spleen as compared to AmB-CNPs and reduced the splenic parasite burden by 75%, whereas AmB-CNPs and AmB alone caused 63 and 47% parasite burden reduction, respectively, in *Leishmania*-infected hamsters [43]. These results were ascribed to the capacity of 4-SO4GalNAc to target resident macrophages. 

As AmB has a poor water solubility, chitosan nanoparticles and Anionic Linear Globular Dendrimers (D) were synthesized and loaded with AmB leading to AK (Amphotericin B-chitosan), and AD (Amphotericin B-Dendrimer) for the treatment of *Leishmania major* CL [44]. The results showed that AmB could be loaded into both these polymers with a loading efficiency of more than 80% and the AmB solubility was enhanced by 80 times for AK and 478 times for AD. In vitro studies revealed a minimum of 90% cell uptake from a slow controlled drug release. No toxicity was observed in vitro and in vivo for both nanodrugs and AK at 10 mg/kg reduced parasite burden to a greater extent (by 83%) than AD did at 50 mg/kg. Further studies are required. 

### 4.2. Paromomycin-Chitosan Nanoformulations

Paromomycin (PM), an aminoglycoside with antiprotozoal properties, is used for the treatment of both VL and CL. However, it has limited intrinsic antileishmanial efficacy, low oral absorption and short half-life demanding innovative strategies to optimize use [45]. Although PM is presently recommended as an ointment (Leishcutan^®^) for the treatment of LCL caused by *Leishmania major*, efforts could be made to improve topical treatments considering the physicochemical properties of this aminoglycoside. One challenge is to enhance the accumulation of enough drug quantity within the dermis where the infected macrophages dwell. Chitosan (CS) was used for the preparation by ionic gelation of a PM-loaded mannosylated CS (MCS) nanoparticles using dextran (PM-MCS-dex-NPs). The particle size of PM-MCS-dex-NPs was estimated at 246 nm, a mannosylation rate of CS at 17%, and an encapsulation efficiency at 83.5%. Acidic media allowed for a better drug release. The mannosylation enhanced the PM uptake by THP-1 cells by about three and four times in comparison to non-mannosylated CS nanoparticles (PM-CS-dex-NPs) and PM solution, respectively. The mannosylation process was also responsible for a seven-times increase in the selectivity index in comparison with the non-mannosylated formulation [45]. Such results highlight the interest of the mannosylation process as an efficient targeted delivery system. 

Mannosylated thiolated chitosan (MTC)-coated PM-loaded poly(d,L-lactide-co-glycolide) (PLGA) nanoparticles (MTC-PLGA-PM) were evaluated for their antileishmanial activity in vitro and in vivo [46]. These particles are spherical with a size of 391 nm. Ex vivo permeation studies showed a 12.7-fold higher permeation of MTC-PLGA-PM compared to free PM. Cellular uptake was markedly increased with the formulation (between 41.98 µg PM/10^6^ cells and 43.11 µg PM/10^6^ cells) by either uninfected or PM-sensitive and -resistant *L. donovani* strains infected J774A.1 macrophages, compared to free PM (between 0.714 µg PM/10^6^ cells and 1.13 µg PM/10^6^ cells). The in vitro antileishmanial activity on *L. donovani* intramacrophage amastigotes was increased by 36-fold with MTC-PLGA-PM compared to free PM. Moreover, the in vivo evaluation of antileishmanial activity revealed a 3.6-fold reduction of parasite burden in *L. donovani* infected BALB/c mice treated with 20 mg/kg/day for 10 days by oral route with MTC-PLGA-PM, compared to free PM. These chitosan-based PM nanoparticles constitute a promising strategy for the treatment of visceral leishmaniasis.

### 4.3. Meglumine Antimoniate-Chitosan Nanoformulations

Mannosylated thiolated chitosan (MTC) and mannosylated thiolated chitosan-polyethyleneimine (MTCE) were also used to incorporate antimonial compounds and analyze their antileishmanial activity [47]. While meglumine antimoniate-loaded nanoparticles inhibited the trypanothione reductase with a K*_i_* at 2 µM, their macrophage uptake was 33.7- and 18.9-fold higher with MTCE and MTC, respectively, in comparison to Glucantime^®^. Moreover, the antileishmanial activity was improved by 14.4- and 7.4-fold with MTCE and MTC, respectively, compared to Glucantime^®^ alone. This formulation designed to improve the therapeutic efficacy of antimonials would have been worthy of in vivo antileishmanial evaluation; however, the probability of development and marketing of a new antimonial formulation in the future is non-existent. 

Microspheres containing different proportions of chitosan, maltodextrin (Glucidex^®^ 19D and 6D), caprylic/capric triglyceride (Labrafac^®^), PEG-40 hydrogenated castor oil (Cremophor RH40^®^) and lecithin (Lemucithin 100^®^) were also used to formulate meglumine antimoniate [48]. All the formulations designed exhibited IC_50_ values between 3.80 to 9.53 µg Sb^V^/mL against *L. infantum* promastigotes, which is considerably lower than the IC_50_ of Glucantime^®^ at 112.26 µg Sb^V^/mL, this last value is unusual as antimonials are known to have very poor activity against promastigotes. An improvement of activity was also observed on intramacrophage amastigotes for two microspheres among the other formulations, with IC_50_ at 31.94 Sb^V^µg/mL and 6.64 µg Sb^V^µg/mL, compared to Glucantime^®^ with IC_50_ values of 176 µg Sb^V^/mL, without cytotoxic effects. Interestingly, substantial activity was obtained with the excipient chitosan on both *L. infantum* promastigotes and amastigotes, with IC_50_ at 112.64 µg chitosan/mL and 100.81 µg chitosan/mL, respectively. Therefore, by lowering the toxic effects of meglumine antimoniate, these chitosan-based formulations would offer new possibilities for the treatment of visceral leishmaniasis, however, the development of new antimonials is not presently encouraged. 

### 4.4. Rifampicin Loaded Nanotransfersomes (NTs) Incorporated in Chitosan Gel

Rifampicin was able to significantly reduce the lesion size in a *L. amazonensis* murine model after an intraperitoneal treatment, but clinical trials in humans were not convincing [49]. Another study reports on 39 patients with cutaneous leishmaniasis provoked by *Leishmania major* who were treated with rifampicin alone or in combination with isoniazid. About 50% of the patients were cured two months after starting the treatment and no significant difference was found between both the treatment regimens [50]. A clinical study including patients affected by *L. tropica* and having received a drug combination rifampicin-omeprazole appeared successful, but this study did not include patients treated with rifampicin alone [51]. Considering these controversial data obtained by parenteral route, a topical application was prepared consisting of rifampicin (RIF)-loaded nanotransferosomes (NTs) incorporated in a chitosan gel [52]. The particle size was 190 nm, with an 83% encapsulation efficiency. Some advantages have been observed by using this formulation such as a three times higher permeation rate than the RIF solution, an enhanced macrophage uptake, and better in vitro and in vivo antileishmanial activities making this formulation worthy of further studies [52].

### 4.5. Curcumin-Chitosan Nanoformulations

Curcumin (Cur) is a natural polyphenolic compound derived from the plant *Curcuma longa* which has been well studied for its anti-cancer, anti-inflammatory, anti-bacterial, and anti-malarial properties [53,54,55,56]. Several studies have reported the antileishmanial activity of Cur, which is presumably mediated through programmed cell death [57,58]. Cur-loaded mannose-functionalized chitosan nanoparticles (Cur-MCN) have been prepared by mannose-conjugated chitosan to better target macrophages in visceral leishmaniasis [59]. This formulation was not cytotoxic for J774A.1 cell line. When evaluated in vivo in a *L. donovani*-infected hamster model, it had the capacity to reduce the spleen parasite burden to a greater extent than unconjugated chitosan nanoparticles [59].

### 4.6. β-. Lapachone-Chitosan

β-Lapachone (β-LP) is a *o*-naphthoquinone obtained from the bark of the Lapacho tree (Bignoniaceae family, *Tabebuia* sp.) which exhibits a wide range of biological activities such as antitumor, antifungal, antibacterial, and antitrypanosomal properties [60]. Besides promoting wound healing, this natural compound has also reported antileishmanial activities on several *Leishmania* species [61,62,63]. However, due to a poor solubility in aqueous solutions, β-LP has to be formulated for the topical treatment of CL. A study focused on a topical treatment of a formulation of β-LP that cures lesions of CL without leaving scars [64]. As lesions are produced by an uncontrolled and persistent inflammatory immune response, the strategy consisted in evaluating β-lapachone (β-LP) loaded in lecithin-chitosan nanoparticles (NP) in order to concentrate the drug into the dermis, where parasites dwell, and also promote an efficient wound healing response through a better permeation through the skin. On the *Leishmania major* infected BALB/c mice model, a topical treatment with β-LP-NP was not able to reduce the parasite burden, but the lesion progression was efficiently stopped [64]. The anti-inflammatory activity was assessed by immuno-histopathological assays in lesions and quantitative mRNA assays in draining lymph nodes. The anti-inflammatory activity of β-LP-NP led to a down-regulation in IL-1β and COX-2 expression and reduced neutrophils infiltration [64]. Such a moderate activity needs further investigations on other leishmaniasis experimental models.

### 4.7. Betulinic Acid-Chitosan

Betulinic acid (BA) is a lupane-type triterpenoid pentacyclic compound that can be isolated from many plant species or obtained from its metabolic precursor, botulin [65]. This natural product presents several biological properties including anti-HIV, antitumor, and antiparasitic activities, in particular against the kinetoplastids *Trypanosoma cruzi* and several species of *Leishmania* [66,67,68]. In *L. donovani*, BA has been shown to induce apoptosis through DNA topoisomerase I and I inhibition [68]. More recently, BA was loaded into nanochitosan (K) to try to improve its therapeutic effects and decrease its adverse effects on a *Leishmania major*-infected BALB/c mice [69]. The synthesized K particles exhibited a size of 102 nm, whereas BA-nanochitosan (BK) had a size of 124 nm and a drug loading efficiency of 93%. The drug uptake of 97% to 98% by cells, and a slow B release without toxicity were significant advantages. The activity of BK in the *L. major* BALB/c mice model at 20 mg/kg by intraperitoneal route for six weeks (alternative days) was shown to enhance the wound healing [69]. Considering these results, BK should be further studied to confirm its interest in the treatment of CL. 

### 4.8. Ursolic Acid-Chitosan

Ursolic acid (UA) is a pentacyclic triterpenoid identified in the epicuticular waxes of apples having anti-inflammatory, anti-bacterial, anti-diabetic, and also antileishmanial activities. UA showed in vitro and in vivo activities in VL and CL experimental models after parenteral and topical applications, respectively [70]. These promising activities led to the development of drug delivery systems to reduce side effects. Therefore, a UA loaded N-octyl-chitosan surface decorated nanostructured lipid carrier system (UA-NLC) was prepared and characterised for the treatment of VL [71]. UA-NLC exhibited a nano size range from 103.7 to 143 nm with 12% drug loading capacity and an entrapment efficiency of 88%. When compared to the macrophage uptake of the UA free form, the formulation had increased uptake of 3 to 12 times depending on the parasite strain. UA-NLC was active in vivo on the *L. donovani*/hamster model by suppressing the parasite burden by 98%.

### 4.9. S-Nitroso-Mercaptosuccinic Acid–Loaded Chitosan Nanoparticles

Nitric oxide (NO)-donors are an attractive option for killing of intracellular parasites. However, they are too chemicaly unstable to be used easily in antileishmanial therapy. Therefore, there is a need for a drug targeting strategy to allow an adapted NO-donor delivery. For this S-nitroso-mercaptosuccinic acid was encapsulated into chitosan nanoparticles (NONPs) for an antileishmanial evaluation on *Leishmania amazonensis* [72]. This formulation was not cytotoxic and a reduction of the number of intramacrophage amastigotes was observed. This formulation is worth further investigations in order to cure cutaneous leishmaniasis.

### 4.10. Chitosan Polymer as a Booster for Drug Efficacy

Previous data from the Paris-Saclay group showed that chitosan particles with typical flat surfaces, called platelets, were able to act as a booster of AmB deoxycholate activity against *Candida albicans* and *C. glabrata* [73,74]. Platelets have been synthesized through a hierarchical self-assembly process between chitosan hydrophobically-modified with oleic acid and α-cyclodextrin in water [75]. In vitro evaluations showed that platelets exhibited intrinsic antileishmanial activity on *Leishmania major* amastigotes, whereas native chitosan (average molecular weight M_w_ = 250,000 g/mol, degree of deacetylation: 85%) did not. Furthermore, an additive combination effect was observed between AmB deoxycholate and chitosan platelets with a Fractional Inhibitory Concentration Index value (FICI) at 1.213. The immunohistochemical and histological analysis of skin lesions of mice infected with *L. major* and treated with this AmB deoxycholate and chitosan platelets combination exhibited a reduction of the inflammatory granuloma and parasite load in comparison with AmB deoxycholate alone [75]. This study underlines the potential interest of a combination of chitosan platelets and AmB as a strategy to cure CL.

Another study focused on the capacity of drug unloaded poly (isobutylcyanoacrylate) nanoparticles coated with chitosan (Cs-NPs) to be active after topical application [76]. No cytotoxicity was observed with this formulation on fully differentiated Caco/TC7 and HT29/MTX cells at 25 µg/mL [77]. In vitro and in vivo evaluation demonstrated an intrinsic antileishmanial activity of Cs-NPs. Daily topical applications on three consecutive weeks on *L. major* infected mice showed that Cs-NPs combined with or without AmB-DOC led to a significant reduction of the parasite load and a partial healing of the lesions that was confirmed by histological studies, in comparison with nontreated mice. The morphology of parasites was affected as shown by transmission electron microscopy: *L. major* promastigotes incubated with Cs-NPs revealed parasitic vacuoles and alterated shape and swelling of mitochondria [76].

### 4.11. Drug Combined Formulations

#### 4.11.1. Amphotericin B-Miltefosine-Chitosan Lipid Nanoparticles

In another biocompatible and biodegradable system, miltefosine (HePC or hexadecylphosphocholine) and AmB have been combined within a chitosan anchored Nanostructured Lipid Carrier (CNLC) leading to HePC-AmB-CNLCs and Tween-80-AmB-CNLCs to get more antileishmanial efficacy [78]. The entrapment efficiency of AmB was about 85% for HePC-AmB-CNLCs with a mean size of particles of 150 nm. The HePC loading efficiency was not determined. The in vitro AmB release from the formulation was slow, corresponding to 65% after a 24 h incubation period for both HePC-AmB-CNLCs and Tween-80-AmB-CNLCs. There was an increased uptake of FITC-HePC-CNLCs over FITC-HePC-NLCs in the J774A.1 cell line by FACS study. Both HePC-AmB-CNLCs and Tween 80-AmB-CNLCs were not haemolytic or cytotoxic. In addition, HePC-AmB-CNLCs were more active than Tween 80-AmB-CNLCs in vitro and in vivo in *L. donovani*- infected hamsters. When administered by the intravenous route, HePC-AmB-CNLCs resulted in an increase of AmB concentration in liver and spleen [78]. Despite the possible interactions between AmB and miltefosine molecules through their respective lipophilic groups and their common affinity to sterols, these results suggested an interesting opportunity to deliver AmB through HePC stabilized chitosan anchored nanostructured lipid carriers with decreased side effects. Moreover, such an approach has the potential to limit drug resistance emergence.

#### 4.11.2. Paromomycin-Selenium-Chitosan Hydrogel

An original selenium derivative, named bis-4-aminophenyldiselenide, exhibited an IC_50_ value of 5.6 µM against *L. major* intracellular amastigotes, justifying further in vivo investigation [79]. Bis-4-aminophenyldiselenide was formulated with paromomycin in chitosan hydrogels and an evaluation of ex vivo permeation and retention in skin layers was carried out by using pig ear skin in Franz diffusion cells. In these conditions, less than 2% to 4% of the diselenide drug was able to penetrate and permeate through the skin, whereas the percentage of paromomycin penetration was in a range from 25% to 60% without important retention within the skin. This formulation was not able to increase lesion regression and reduce parasite burden on a *L. major* infected BALB/c mice model as the result of its inability to deliver the selenium derivative [79].

#### 4.11.3. Antimony-Titanium-Chitosan

Nanoassemblies of chitosan-titanium dioxide (TiO_2_) nanoparticles (NPs) loaded with Glucantime^®^ were designed for getting an advantage over their possible additive effects against *L. major* [80]. The nanoassemblies with the optimal ratio by using 12.5 mg Glucantime^®^, 25 mg chitosan, and 6 mg TiO_2_ NPs were prepared and characterized for their physico-chemical characteristics, and mainly loading and release efficiency of drug from nanoassemblies. When evaluated in vitro against *L. major* promastigotes, TiO_2_ NPs exhibited similar activity as those with Glucantime^®^ alone. However, nanoassemblies were able to decrease both promastigote and intramacrophage amastigote proliferation by 13 and four times, respectively, compared to Glucantime^®^ alone, after a three-day exposure at 50 µg/mL final concentration [80].

## 5. Immunomodulatory Effect of Chitosan

Chitin, as chitosan, is considered to be able to stimulate the immune system. A comparative study was therefore performed considering the protective effects of microparticles (MP) of both these polymers on a *L. major* BALB/c mouse model [81]. The MP size was less than 40 µm. After a subcutaneous infection of BALB/c mice with promastigotes, chitin or chitosan MPs were administered by subcutaneous route over two weeks with two-day intervals. At the autopsy, the lesion sizes were 10 times and five times smaller after chitin and chitosan administration, respectively, in comparison with the lesion size of control group. In the same manner, the parasite load of the lymph nodes was more reduced by chitin MPs than by chitosan MPs [81]. In addition, chitin MPs enhanced IL-10 and TNF-α productions whereas chitosan MPs did not. This study clearly demonstrates the advantage of chitin over chitosan as an immunomodulator in the *L. major* murine model. 

In order to get an immunoadjuvant effect to AmB therapy, AmB loaded pluronic F127 (PF 127) micelles were coated with chitosan (Cs-PF-AmB-M), giving them macrophage targeting properties. This formulation was about eight to 10-fold less cytotoxic than AmB suspension [82]. Flow cytometry revealed that Cs-PF-FITC-M was more internalized by J774A.1 macrophages than PF-FITC-M. In vitro and in vivo antileishmanial activities of Cs-PF-AmB-M were encouraging with a stimulation of a Th1 immune response. The intravenous administration of the formulation had no effect on blood urea nitrogen and plasma creatinine levels and the pharmacokinetic data demonstrated that Cs-PF-AmB-M was able to circumvent the classical AmB nephrotoxicity [82]. From these results, Cs-PF-AmB-M appears to be a possible formulation candidate combining both immunological and therapeutic targets, without acute toxicity for the treatment of visceral leishmaniasis.

Nanometric amphotericin B (AmB)-encapsulated chitosan nanocapsules (CNC-AmB) have been prepared using a polymer deposition technique mediated by nanoemulsion template fabrication [83]. The presence of chitosan was beneficial to CNC-AmB stability in the presence of protein and Ca(2+). In vitro, CNC-AmB was less toxic to both J774A.1 cells and erythrocytes than conventional AmB formulations such as Fungizone^®^ and AmBisome^®^. CNC-AmB was active both in vitro on *L. donovani* intramacrophage amastigotes with an IC_50_ value at 0.2 µg/mL and in vivo on *L. donovani*-infected hamsters with 86% splenic parasite burden reduction after an intraperitoneal treatment on five consecutive days, making a total dose of 5 mg AmB/kg of hamster body weight. These activities were positively correlated with the upregulation of tumor necrosis factor alpha (TNF-α), interleukin-12 (IL-12), and inducible nitric oxide synthase and with the downregulation of transforming growth factor β (TGF-β), IL-10, and IL-4 [83]. CNC-AmB can therefore be considered as an immunoadjuvant chemotherapy delivery system most likely cheaper than AmBisome^®^.

## 6. Chitosan and Chitin for Antileishmanial Vaccines

There are a number of *Leishmania* vaccines in development [84], all with limitations but some now making progress in clinical trials [85]. Chitosan has potential roles in vaccine development both as a delivery vehicle for requisite antigens and as an adjuvant, on which there have been few studies, for leishmaniasis [86]. 

### 6.1. Leishmania Antigens Encapsulated in Chitosan Nanoparticles

A first generation *Leishmania* vaccine consisting of a whole *Leishmania* lysate antigen (WLL) and soluble *Leishmania* antigens (SLA) were encapsulated in chitosan nanoparticles and studied in BALB/c in a murine model of leishmaniasis at the level of immune response [87]. An ionic gelation method was used to load chitosan nanoparticles with antigens, according to a polymer/antigens optimised ratio. The formulation was characterized and used for BALB/c mice immunization by subcutaneous route versus antigens alone three times with two-week intervals. The results were disappointing, as a mixed Th1/Th2 immune response was obtained, whereas a Th1-type immune response was expected [87].

### 6.2. Leishmania Superoxide Dismutase Loaded Chitosan Nanoparticles

A study reports on the loading of recombinant *Leishmania* superoxide dismutase (SODB1) onto chitosan nanoparticles [88]. These particles were prepared by using ionotropic gelation and were physicochemically characterised. Then, a study was performed on the *L. major* BALB/c mice model through immunization by the subcutaneous route in three doses at three weekly intervals. The IgG2a and IgG1 analysis in blood, performed three weeks after the last injection, indicated a significant increase of the IgG2a/IgG1 ratio, whatever the administration scheme with one or three SODB1 nanoparticles doses. These encouraging results suggest that SODB1 loaded chitosan nanoparticles are able to stimulate the cell-mediated Th1 immunity, suitable for the development of nanovaccines [88].

## 7. Conclusions

Leishmaniases mainly affect poor populations, therefore the therapy/vaccine strategies should be both reliable and cheap, making them affordable for patients and populations. It is expected that any particulate formulation, such as AmBisome^®^, significantly increases the treatment cost, and if the formulations are more sophisticated, the probability of pharmaceutical development will be jeopardized.

In this context, the first advantages of chitosan are its ease of production at low cost and its biodegradability. This review has highlighted interesting data, paving the way to further investigations on leishmaniasis both in therapeutic and vaccine research. However, despite the number of data collected, it was not possible to highlight the most relevant physico-chemical characteristics of the formulations responsible for the best in vitro selectivity index and in vivo activity, as many parameters, i.e., the nature of the polymer, the particle size, the zeta potential, the loaded drugs, and the different strains and protocols used for their biological evaluation are intimately entangled. Further studies focused only on AmB, for example, would be useful for a strict comparison between the most promising formulations by using the same in vitro and in vivo models of experimental leishmaniasis. The second advantage of chitosan is its intrinsic antileishmanial activity. Thus, when combined with another drug, it acts as a booster and, to some extent, it can also be considered as an active principle for drug combination. Few studies have focused on the mechanism of action of chitosan on *Leishmania*. It is known that *Leishmania* sp. expresses chitinase activity thought to be important in parasite-sandfly interactions and transmission of the parasite to the vertebrate host [89]. Even if chitinases have the unique ability to hydrolyse GlcNAc-GlcNAc bonds, making these enzymes capable of hydrolysing chitin, they are able to hydrolyse, to some extent, partially acetylated chitosan as well [90]. Thus, the importance of leishmanial chitinases in the mechanism of action of chitosan is worthy of further consideration if GlcNAc oligomers exhibit intrinsic antileishmanial activity.

Significative efforts on chitosan research have been carried out for a decade in antileishmanial chemotherapy by association with drugs, and mainly AmB, as this drug is a star in antileishmanial therapy. However, improvements in activity are required via different strategies including various chitosan formulations. Thus, several parameters such as the degree of deacetylation, the particle size, and the administration route gave various possibilities and promising perspectives of chitosan for therapies and vaccines.

## Supplementary Materials

The following are available online, Table S1: Physico-chemical characteristics of the chitosan-based antileishmanial formulations. ND: Not determined, Table S2: Biological activity of the chitosan-based antileishmanial formulations. ND: Not determined; AP: Active principle
